# The impact of a faculty development programme for health professions educators in sub-Saharan Africa: an archival study

**DOI:** 10.1186/s12909-015-0320-7

**Published:** 2015-03-03

**Authors:** José M Frantz, Juanita Bezuidenhout, Vanessa C Burch, Sindi Mthembu, Michael Rowe, Christina Tan, Jacqueline Van Wyk, Ben Van Heerden

**Affiliations:** 1 Faculty of Community and Health Sciences, University of the Western Cape, Bellville, South Africa; 2 Department of Interdisciplinary Health Sciences, Faculty of Medicine and Health Sciences, Stellenbosch University, Tygerberg, South Africa; 3 Department of Medicine, Faculty of Health Sciences, University of Cape Town, Rondebosch, South Africa; 4 KwaZulu-Natal College of Nursing, Pietermaritzburg, KwaZulu-Natal South Africa; 5 Medical Education & Research Development Unit, Faculty of Medicine, University of Malaya, Kuala Lumpur, Malaysia; 6 College of Health Sciences, University of KwaZulu-Natal, Durban, South Africa

**Keywords:** Faculty development, Kirkpatrick framework, Evaluation

## Abstract

**Background:**

In 2008 the sub-Saharan FAIMER Regional Institute launched a faculty development programme aimed at enhancing the academic and research capacity of health professions educators working in sub-Saharan Africa. This two-year programme, a combination of residential and distance learning activities, focuses on developing the leadership, project management and programme evaluation skills of participants as well as teaching the key principles of health professions education-curriculum design, teaching and learning and assessment. Participants also gain first-hand research experience by designing and conducting an education innovation project in their home institutions. This study was conducted to determine the perceptions of participants regarding the personal and professional impact of the SAFRI programme.

**Methods:**

A retrospective document review, which included data about fellows who completed the programme between 2008 and 2011, was performed. Data included fellows’ descriptions of their expectations, reflections on achievements and information shared on an online discussion forum. Data were analysed using Kirkpatrick’s evaluation framework.

**Results:**

Participants (n=61) came from 10 African countries and included a wide range of health professions educators. Five key themes about the impact of the SAFRI programme were identified: (1) belonging to a community of practice, (2) personal development, (3) professional development, (4) capacity development, and (5) tools/strategies for project management and/or advancement.

**Conclusion:**

The SAFRI programme has a positive developmental impact on both participants and their respective institutions.

## Background

More than two decades ago, Shugars et al. highlighted the need for health professions education (HPE) “to be a part of the solution to the problems facing health care” [1]. They suggested that in order to achieve lasting change health professions educators must “act as advocates for change at the levels of the individual, school, the university, the health professions, the government, and the public …” In 2002, the World Health Organisation in the African region highlighted the need for “strategic partnerships in education and health in Africa” [2] and endorsed seven critical and interrelated issues affecting the health workforce. They also identified two key areas in the domain of HPE that were in need of immediate attention. These included:

*(a) “The need to ensure the relevance of education and training of health professionals to the health needs of the population served”,* and

*(b) “The importance of forging new partnerships between the health and education sectors and of continuing professional development of the health workforce”.*

A commissioned report published in *The Lancet* in 2010 presented the results of a comprehensive investigation into the current global status of HPE, highlighting the range of challenges that have emerged as a result of the increasing complexity of healthcare systems [3]. The authors of this report identified two key issues with regard to HPE in the 21st century: (1), the need for transformative learning to graduate leaders and change agents, and (2) recognition of the interdependence of role players involved in healthcare and HPE. Both a report from the World Health Organisationt [2], as well as *the Lancet* article emphasised that the primary aim of HPE should be to address the health needs of the populations and health systems we serve. In other words, the competencies that are developed by medical students should be “adapted to local contexts and be determined by national stakeholders” [3]. This highlighted the urgency to find appropriate strategies to address a wide range of HPE needs in Africa.

Faculty development is considered an essential component of the academic success of individual faculty members and their institutions. It has also been identified as an appropriate strategy to address issues such as poorly developed teaching strategies and a lack of research in HPE [4]. An important role player in the process of achieving these goals in developing countries is the Foundation for Advancement of International Medical Education and Research (FAIMER), a USA-based non-profit organisation affiliated with the Educational Commission for Foreign Medical Graduates (ECFMG), which was founded in 2001 [5]. FAIMER offers an education and leadership faculty development programme, which aims “to strengthen medical education and to build a sustainable discipline of medical education in developing countries” [6]. Initially the fellowship programme included two residential sessions in the USA, but FAIMER has established six equivalent fellowship programmes hosted by regional institutes in three world regions: Asia (India and China), South America (Brazil) and sub-Saharan Africa (South Africa). The African programme was established in 2008, and is hosted by the Sub-Saharan African FAIMER Regional Institute (SAFRI) based in Cape Town, South Africa.

The SAFRI faculty development programme focuses on developing African health professions educators as leaders, teachers, scholars and advocates for change at multiple levels, including the individual, school, university and the health professions. Each year FAIMER awards 16–18 fellowships to sub-Saharan African health professions educators to attend the SAFRI programme, which includes (1) three residential sessions of five to nine days each, (2) a distance learning component and (3) the design and completion of an education innovation project which focuses on improving HPE in the home institution of each fellow (Table 1).

**Table 1:** **Example of the SAFRI two-year fellowship programme**

Sessions	Topics for discussion	Time frame
Distance learning session 1	Research and evaluation:	10 weeks in Year 1
	•Literature review and writing a research question	
On-site session 1	•Welcome	7 days in Year 1
	•Team work	
	•Understanding your leadership style	
	•Project planning and design	
	•Change management	
	•Conflict management	
	•21st century leadership	
	•Sustaining change	
Distance Learning session 2	Leadership:	10 weeks in Year 1
	•Leadership in Health Professions Education	
	•Understanding and managing self	
	•Understanding and managing others	
	•Team building	
Distance Learning session 3	Student Assessment:	10 weeks in Year 1
	•Blue printing	
Off-site session	Project implementation	6-9 months in Year 1
	Project write up	
On-site session 2	•Welcome and re-entry	5 days in Year 2
	•Programme evaluation	
	•Electronic portfolio development	
	•Poster presentation of project	
	•Scholarship of education	
	•7-minute conference presentation	
	•Curriculum design	
	•How to set up an OSCE workshop	
	•Feedback and portfolios	
	•Interactive teaching	
	•Work-based learning	
	•Setting up a faculty development workshop	
On-site session 3	•Welcome and re-entry	4 days in Year 2
	•Blueprinting workshop	
	•Writing MCQs workshop	
	•Article writing clinic	
	•Attendance of the South African Association of Health Educationalists’ Conference	

During the residential sessions all the teaching activities are focused around interactive small group activities focusing on three main themes: (1) leadership and change management skills, (2) project management tools and (3) HPE, as seen in Table 1. During the first residential session, dedicated time is allocated to the task of writing an education innovation study proposal. Since most of the fellows entering the programme are novice researchers in HPE, they are guided and supported by a community of African health professions educators who teach, practise and conduct HPE-related research in a wide range of sub-Saharan African countries. During the first residential session participants are also taught various research and project management strategies to ensure successful completion of their research projects in their respective home institutions.

During the second year of the programme, fellows focus on the scholarship of HPE and engage in three scholarly activities that are embedded within, and based upon, the educational research project completed during the first year of the fellowship. First, they present their project work at the annual SAFRI Poster Day attended by all the SAFRI faculty teaching in the programme as well as invited guests, including deans and deputy deans of local or other universities where the fellows work. Second, they submit an abstract to present their work as a poster or oral presentation at the annual HPE conference hosted by the South African Association for Health Educationalists (SAAHE) in South Africa each year. Third, on site faculty support is provided during the final residential session, which runs concurrently with the SAAHE conference, to write up their work as a manuscript for publication in an HPE journal focusing on the needs of developing countries. The African Journal of Health Professions Education, a journal focused on advancing the scholarship of HPE in Africa, has been a suitable platform for a number of publications by both SAFRI faculty and fellows.

While new knowledge and skills are important early outcomes of the fellowship programme, an important long term outcome is access to, and participation in, a community of practice of health professions educators in an African healthcare context and the development of skills that will assist SAFRI fellows in becoming change agents as described in the papers published by the WHO and *The Lancet* [2,3].

While many faculty development programmes have been successfully implemented, there remains a need to evaluate their impact in order to justify the time, effort and resources that are required to run these programmes. While there are many ways of of critically appraising the impact of faculty programmes like the SAFRI programme, the Kirkpatrick model of evaluation is an attractive option in resource-constrained settings because it is not resource-intensive or complex to perform [7]. This framework essentially consists of four levels of evaluation:

1. Participant reaction: the expectation(s) of participants in the programme;
2. Participant learning: any new knowledge, skills and abilities acquired by participants during the programme;
3. Transfer of knowledge: extent to which participants’ transfer what they have learnt to their work context, both during and after completion of the programme;
4. Productivity gains: the impact of the programme on the department /organisation where the participant is employed.

### Aim

The aim of this study was to determine participants’ perceptions of the impact of the SAFRI fellowship on their personal and professional development using Kirkpatrick’s evaluation framework.

## Methods

### Research design

This study was conducted using a retrospective archival research design. SAFRI records and documents were reviewed to evaluate the fellowship programme as it relates to the development of HPE scholarship capacity in Africa. Archival analysis refers to an observational research method whereby the researcher examines a set of accumulated documents or archives [8]. This form of analysis is attractive because it is cheap and deals only with recorded information.

### Sample

Information was obtained for all of the fellows (N=61) who participated in the SAFRI programme during the period 2008–2011.

### Data collection methods

At the beginning of each residential session, participants were instructed to ask three other new fellows three focussed questions, record their answers on flipcharts and then present the information about each interviewee to the bigger group. The questions were designed to capture the fellows’ aspirations and expectations at the start of the programme. This information was used to address the first level of the Kirkpatrick framework.

Between the first and second residential session, fellows were required to participate in an online forum where HPE issues were discussed and experiences were shared with other fellows and faculty. This information addressed the second and third levels of the Kirkpatrick framework.

At the start of the second residential session, one year after commencing the programme, fellows were asked to repeat the exercise done at the start of the first session. Questions for the activity were designed to capture the impact of the programme on their personal and professional development during the past year, i.e. one year after joining the programme.

This information recorded by the fellows on flipcharts, the main ideas that emerged were highlighted. Kirkpatrick’s second and third levels were evaluated through informal comments on the online medium during the programme. Comments made by fellows one year after commencing the fellowship, at the second on-site session when participants evaluated the impact of the programme on their personal and professional development. This information addressed the final level of the Kirkpatrick framework.

The data were transcribed onto Excel spreadsheets and analysed qualitatively.

### Data analysis

The information from the various data sources was captured by one author (CT) and was then circulated to a group of reviewers (JF, SM, JB) who independently colour-coded the information to identify similar thoughts and ideas. The coders were instructed to use the Kirkpatrick framework as a guide and code the various responses according to the Kirkpatrick framework levels. Once this was done, the various categories embedded within each level were identified. The colour codes were compared across the responses to identify relations within the data and to categorise key concepts, which were then shaped into a thematic framework. The identified themes were discussed by the group until consensus was reached. A further trustworthiness check was done by searching the documents for content that could disprove the primary findings. All themes are presented with quotations to ensure the trustworthiness of the information provided.

Permission to conduct the study was obtained from the Research Ethics Committee of the University of the Western Cape (Project no: 11/3/15). At the start of each intake, fellows granted SAFRI permission to use archived information collected during the programme. Information was presented in a manner, which ensured that confidentiality of all responses was maintained.

## Results

### Participant demographics

A total of 64 health professions educators representing 11 African countries participated in the programme between 2008 and 2011. Of these, three did not complete the programme. A summary of the demographics of the participants is presented in Table 2.

**Table 2:** **Demographics of the participants**

Variable	2008	2009	2010	2011	TOTAL
**Mean Age**	50.5	40.3	42.9	41.8	42.1
**Gender**	**16**	**15**	**16**	**17**	**64**
Female	11	5	9	9	34
Male	5	10	7	8	30
**Profession**	**16**	**15**	**16**	**17**	**64**
Medicine	5	8	6	11	30
Nursing	2	2	4	1	9
Dentistry	0	1	1	2	4
Allied Health	5	1	3	2	11
Education	4	3	2	1	10
specialist					
**Country**	**16**	**15**	**16**	**17**	**64**
South Africa	13	5	8	4	30
Uganda	2	5	4	3	14
Sudan		1	2	2	5
Zambia	1	1	1	1	4
Zimbabwe				2	2
Malawi		2			2
Mozambique		1	1		2
Tanzania				2	2
Nigeria				1	1
Madagascar				1	1
Botswana				1	1

### Expectations of the fellowship

Participants reported that they hoped participation in the SAFRI programme would improve their knowledge of HPE, research methodology, faculty development and curriculum development. A second expectation was that there would be an improvement in their leadership, project management and research skills as well as in conducting student assessment. They also valued the opportunity to network with others (face-to-face and online) with similar interests, and share resources.

### Impact of the programme

Five key themes, representing all four levels of the Kirkpatrick framework, emerged from the fellows’ reflections on the impact of the programme (see Table 3). These were grouped as: (1) belonging to a community of practice, (2) personal development, (3) professional development, (4) use of tools and strategies for project management and/or advancement, and (5) capacity development. Within each theme there were categories that highlighted the impact of the SAFRI programme.

**Table 3:** **Themes and sources of data**

Kirkpatrick levels	Sources of data	Participants’ experiences	Categories	Themes
*Level 1: Participants’ reaction*	*On-site sessions*	Participants felt the SAFRI fellowship allowed them the opportunity to share their experiences and learn from others as well as build up a network of colleagues	Sharing ideas	**Community of Practice**
			Exchanging information	
	*Electronic mailing list*		Networking	
			Togetherness	
	*Online discussion forum*		Network of colleagues	
*Level 2:* *Participants’ learning*	*Check in at beginning of every session*	Participants identified that the SAFRI experience allowed for personal growth and created opportunities for development	Growth	**Personal Development**
			New opportunities	
			New interests	
			Recognition	
			Paradigm shift	
	*Short interviews with focussed questions*			
*Level 3:* *Extent to which participant transferred knowledge to new situations*	*Check in at beginning of every session*	Participants made shifts in their careers in terms of research and identified opportunities in publications, awards and conference attendances	Increased publications	**Professional Development**
			Educational scholarship awards	
	*Short interviews with focussed questions*		Conference attendances and presentations	
			National Research Foundation rating research development	
			Recognised research roles	
*Level 3:* *Extent to which participant transferred knowledge to new situations*	*On line discussions*	Participants were clearly using the tools they had learnt during the fellowship in their work environment	Elevator speech	**Use of Tools**
			Assessment tools	
			MBTI	
			Force field analysis	
			Gantt chart	
	*Feedback from projects*			
	*Feedback post one year*			
*Level 4:* *Behaviour change*	*On line discussions*	Participants shared their knowledge with others and aimed to start activities that would build capacity in their home institutions	Talk to other people	**Capacity Development**
			Education of interest groups	
	*Check in at the beginning of every session*		Building capacity of newly appointed academics	
	*Short interview with focussed questions*			

### Community of practice

This theme was classified as an example of the Level 1 category of the Kirkpatrick framework as it was the participants’ reaction to how they experienced the SAFRI fellowship. The participants indicated that the SAFRI experience had allowed them to share ideas and exchange information and provided opportunities for networking and building relationships. Participants also reported that access to the immediate network had expanded because meeting one new person connected them to the network of the new person. The following are excerpts from the comments of some participants:

*“International collaboration and fellowship engendered by SAFRI helped to promote my feelings on African development.”*

*“… but far more important has been the opportunity through SAFRI to be a member of an expanding community where I can continue to contribute and be challenged in so many exciting ways …”*

### Personal development

This theme highlighted Level 2 of the Kirkpatrick framework in terms of the participants’ learning experiences. Participants emphasised that the SAFRI experience had promoted their personal growth as researchers and academics, while also affording them opportunities to create new interests. The personal recognition obtained from the process was satisfying to the participants. Some of the participants indicated that:

*“The biggest growth for me was a fifty-fold increase in my self-confidence regarding my instincts.”*

*“Understanding different personalities has empowered me to deal with other people.”*

### Professional development

This theme addressed Level 3 of the Kirkpatrick framework; the extent to which participants transferred knowledge to new situations. Participants found that the SAFRI experience had facilitated an increase in their educational scholarship, including peer-reviewed publications, educational scholarship awards, conference attendance and presentations, national research ratings, research development and recognised research roles. It emerged that participants embraced shifts in their careers in terms of research as well as academic promotions.

*“My new role as Departmental Research Committee Chair was quite challenging as several new changes had to be made. My SAFRI experience contributed immensely to my success in this role till now …”*

*“Ever since I joined this family, I became so interested in education research and I have so far published six papers in this research.”*

### Tools and strategies for project management and/or project advancement

This theme also addressed Level 3 of the Kirkpatrick framework; the extent to which fellows transferred knowledge to new situations and/or a changed in their professional behaviour /practice. Participants reported that the SAFRI experience had taught them specific skills and introduced them to a range of formative and summative assessment tools as well as several management tools, including an “elevator speech”, the Myers-Briggs Type Indicator (MBTI), force field analysis and Gantt charts.

A brief explanation of the respective management tools is provided here. An elevator speech teaches participants to summarise an idea, product, service or event that they want to get across in the time span of an elevator ride (approximately 30 to 120 seconds’ in duration). The MBTI assessment uses a psychometric questionnaire to measure psychological preferences for perceiving the world and making decisions. Force field analysis provides a useful decision-making technique, using an analysis of the forces for and against a proposed change of practice. Participants were also taught to use Gantt charts, commonly employed in project management, to illustrate the project schedule by displaying activities along a time line or schedule. Participants provided enthusiastic reports of the use of the various management tools in their work environment. These are some comments from the participants:

*“The Gantt chart was most useful in negotiating my work load for this year, so that I could add more value to the responsibilities that I do take on.”*

*“I use the elevator speech often; even five days ago I had a talk with the Dean …”*

### Capacity development

This theme addressed Level 4 of the Kirkpatrick framework in which participants become active change agents and change their behaviours in their respective professional settings. Participants reported that the SAFRI experience had given them the opportunity and skills to communicate and engage with other academics and education interest groups. They were also able to facilitate the professional development of newly appointed academic staff. Participants shared their knowledge with others and aimed to start activities that would build capacity in their home institutions. Some comments from the participants illustrate the changes made

*“Introducing research into teaching and learning among junior staff …”*

*“… started an Education Research Interest Group … and there are a number of enthusiastic staff working towards publication …”*

## Discussion

This study reports on the impact of an international faculty development programme, which specifically addresses the leadership, teaching and scholarship needs of a diverse range of health professions educators living and working in sub-Saharan Africa. The major themes that emerged from the data were the development of communities of practice and personal and professional development in the areas of leadership, HPE and scholarship using the management and research tools discussed and used during the fellowship programme.

### Community of practice

While CoPs in health care vary greatly, they share four essential characteristics: social interaction among members, knowledge sharing, knowledge creation, and identity building [9]. The most important feature of the communities of practice that develop in this programme is the diversity of countries, professions and levels of career development of the participants. It is clear from the participants’ responses that becoming part of such a community was an important reason for applying to the programme. Participants sought not only to learn from others, but also to make their own contributions to the community. The programme clearly met their expectations and provided opportunities for the sharing of ideas and collaboration, even across national boundaries. At SAFRI, CoPs emerged as groups of health professions educators who share concerns, problems, or passion about topic(s), and who deepen their knowledge and expertise in specific areas by interacting on an on-going basis [10]. This orientation to capacity building is itself embraced as integral to the academic programmes and is at the core of the SAFRI initiative. The central theme in a CoP, as observed in SAFRI, was learning through a process of acculturation into a community, gradually assuming additional roles and responsibilities with the socio-cultural practices of this particular community [11].

### Personal and professional development

Personal and professional growth, key to the development of health professions educators, begins when an individual joins a new group or community with the prospect of becoming a full participant [12]. The professional development of SAFRI fellows was facilitated by participation in a wide range of activities and the roles they assumed within the SAFRI community and other communities of practice within their home institutions and further afield. In the current study, participants recognised the benefits of the programme in terms of its intrinsic and extrinsic value to them. They ascribed the growth in their personal and professional identities, to new experiences that challenged their established points of view. It was evident that the diversity of experiences, nationality, culture and professions, present in each intake of SAFRI fellows, contributed to the strong sense of personal development and group identity that developed during the residential sessions. The contribution that diversity makes to strengthen communities has been previously reported [13]. In this study it was also evident that the diverse experiences of fellows strongly influenced the development of awareness and insight into HPE as a discipline, and the important role they could play. In their personal journeys as health professions educators, participants also gained insight into the broad spectrum of health professions educators and ways in which they could interact as part of multiprofessional healthcare teams. This change in beliefs, attitudes and understanding about their respective and inter-related roles was evidence of their professional growth during the programme [14,15].

In addition to personal changes participants also described the SAFRI programme as an opportunity to advance their professional standing in their fields of study and practice at their home institutions. Fellows reported job promotions and increased research productivity as evidenced by peer reviewed publications and conference attendance and presentations. A platform to share and compare learning and ways of understanding others, for the purpose of personal professional development, is well aligned with the collegial aspects of, and discipline approaches to, faculty development [16]. Collaborative work in collegial groups enabled individuals to examine their thinking about their own teaching and critique the perspectives and understandings of their colleagues [16].

According to Jarvis-Selinger et al. [12], reflection on the relationship between social roles, professional identity, and individual competence specific to a particular community of practice is “critical to the development of a person”. The key findings of this evaluation of the impact of the SAFRI programme, using the framework described by Kirkpatrick, highlights this interplay between the individual, professional and community roles of health professions educators.

### Tools and strategies for project management/advancement

Tools, used as part of the programme’s research and teaching activities also contributed to the personal and professional development of the participants. Throughout the programme, they were introduced to a range of outcome measures, frameworks and models that can be used to improve research and teaching practices. Participants reported regular use of these tools as part of their daily work, indicating that they clearly derived value from them.

### Capacity development

Many of the participants indicated that the SAFRI programme was not only of personal benefit but that it also provided fellows with opportunities to contribute to the development of their peers. In particular, participants spoke about being able to assist less experienced colleagues on returning to their own institutions. In addition fellows spoke of the poorly developed culture of scholarship in teaching and learning, and HPE-related research in their home institutions, and described how their experiences in the SAFRI programme enabled them to return home and begin (or reinforce) a culture to assist others in developing in these areas. Successful faculty development programmes require that they be driven from within, which is the only way to ensure that they are sustainable, systematic, realistic and collaborative [17].

### Limitation of the study

Although the methods used in this study are of a self-report nature and we acknowledge this limitation, the information provided at the various stages of the programme provides evidence of translation of some of the knowledge acquired into action. While archival studies, which only evaluate documented activities or outcomes, are considered to be fairly objective researchers should be aware that the documents may be unrepresentative, selective, subjective and possibly even deceptive [8]. In this study the issue of inaccurate archival data was not entertained because the scholarship outputs of the fellows, including posters, conference presentations and peer-reviewed publications, provided sufficient concrete evidence of the impact of the programme from an academic perspective.

The use of self-reported data may be considered a second limitation of this study. However, tangible evidence of the scholarship activities of the fellows (conference presentations and publications) validated the self-reported data describing improvements in knowledge and skills, changed attitudes and new insights regarding HPE, as well as use of the new knowledge and skills to facilitate the development of colleagues both within the programme as well as in their home institutions.

## Conclusion

The data presented in this paper confirm the hypothesis that the SAFRI programme has a positive impact on the personal and professional development of health professions educators from a diverse range of professions, countries and universities in sub-Saharan Africa. In order to have such an impact, programmes geared at health professions educators must be cogniscent of the local context in which participants work, teach and pursue the scholarship of HPE. Furthermore, the diversity demonstrated in the SAFRI programme demands a broad, context-sensitive knowledge of HPE as well as excellent teaching and research skills to run such programmes [18,19]. The main areas of personal and professional development identified in this study were the development of communities of practice and capacity development as health professions educators and scholars of HPE Finally, this paper provides data in support of the positive impact of faculty development programmes such as the one offered by the sub-Saharan African FAIMER Regional Institute.
